# Engineering *Yarrowia lipolytica* to Produce Glycoproteins Homogeneously Modified with the Universal Man_3_GlcNAc_2_ N-Glycan Core

**DOI:** 10.1371/journal.pone.0039976

**Published:** 2012-06-29

**Authors:** Karen De Pourcq, Petra Tiels, Annelies Van Hecke, Steven Geysens, Wouter Vervecken, Nico Callewaert

**Affiliations:** 1 Unit for Medical Biotechnology, Department for Molecular Biomedical Research, VIB, Ghent, Belgium; 2 L-Probe, Department of Biochemistry and Microbiology, Ghent University, Ghent, Belgium; 3 Department of Biomedical Molecular Biology, Ghent University, Ghent, Belgium; 4 Oxyrane Belgium, Ghent, Belgium; Technical University of Denmark, Denmark

## Abstract

*Yarrowia lipolytica* is a dimorphic yeast that efficiently secretes various heterologous proteins and is classified as “generally recognized as safe.” Therefore, it is an attractive protein production host. However, yeasts modify glycoproteins with non-human high mannose-type N-glycans. These structures reduce the protein half-life *in vivo* and can be immunogenic in man. Here, we describe how we genetically engineered N-glycan biosynthesis in *Yarrowia lipolytica* so that it produces Man_3_GlcNAc_2_ structures on its glycoproteins. We obtained unprecedented levels of homogeneity of this glycanstructure. This is the ideal starting point for building human-like sugars. Disruption of the *ALG3* gene resulted in modification of proteins mainly with Man_5_GlcNAc_2_ and GlcMan_5_GlcNAc_2_ glycans, and to a lesser extent with Glc_2_Man_5_GlcNAc_2_ glycans. To avoid underoccupancy of glycosylation sites, we concomitantly overexpressed *ALG6*. We also explored several approaches to remove the terminal glucose residues, which hamper further humanization of N-glycosylation; overexpression of the heterodimeric *Apergillus niger* glucosidase II proved to be the most effective approach. Finally, we overexpressed an α-1,2-mannosidase to obtain Man_3_GlcNAc_2_ structures, which are substrates for the synthesis of complex-type glycans. The final *Yarrowia lipolytica* strain produces proteins glycosylated with the trimannosyl core N-glycan (Man_3_GlcNAc_2_), which is the common core of all complex-type N-glycans. All these glycans can be constructed on the obtained trimannosyl N-glycan using either *in vivo* or *in vitro* modification with the appropriate glycosyltransferases. The results demonstrate the high potential of *Yarrowia lipolytica* to be developed as an efficient expression system for the production of glycoproteins with humanized glycans.

## Introduction

There is increasing demand for efficient expression systems for the economical production of biopharmaceuticals. The properties of recombinant biopharmaceutical proteins can be fine-tuned by manipulating the glycan structures attached to them. However, versatile production methods for producing specific glycoforms are few and involve mostly laborious *in vivo* pathway engineering.

To rapidly generate different glycoforms of a particular biopharmaceutical for functional studies and subsequent production, it would be valuable to have a microbial expression system that produces N-glycoproteins homogenously modified with the Man_3_GlcNAc_2_ N-glycan core. This core is common to all mammalian N-glycan structures, and any complex type N-glycan can be built *in vitro* on this core using the appropriate glycosyltranferases and sugar-nucleotide donors. However, no convenient expression system producing this Man_3_GlcNAc_2_ core is currently available. Our objective was to engineer the yeast *Yarrowia lipolytica* for this purpose.

Yeasts combine the ease of genetic manipulation and up-scaling of microbial cultures with the ability to secrete and modify proteins with the major eukaryotic post-translational modifications. *Saccharomyces cerevisiae* and the methylotrophic yeasts *Pichia pastoris* and *Hansenula polymorpha* are the most frequently used yeast hosts for recombinant protein production, but there is growing interest in the dimorphic yeast *Yarrowia lipolytica*. This yeast can grow to high cell density on long-chain fatty acids. The promoters of acyl-CoA oxidase (POX) genes are strongly induced on this carbon source and are therefore used to drive heterologous gene expression. Moreover, *Y. lipolytica* has long been used for the production of lipases for the agro-food industry and is therefore classified as GRAS (generally regarded as safe).

**Figure 1:**
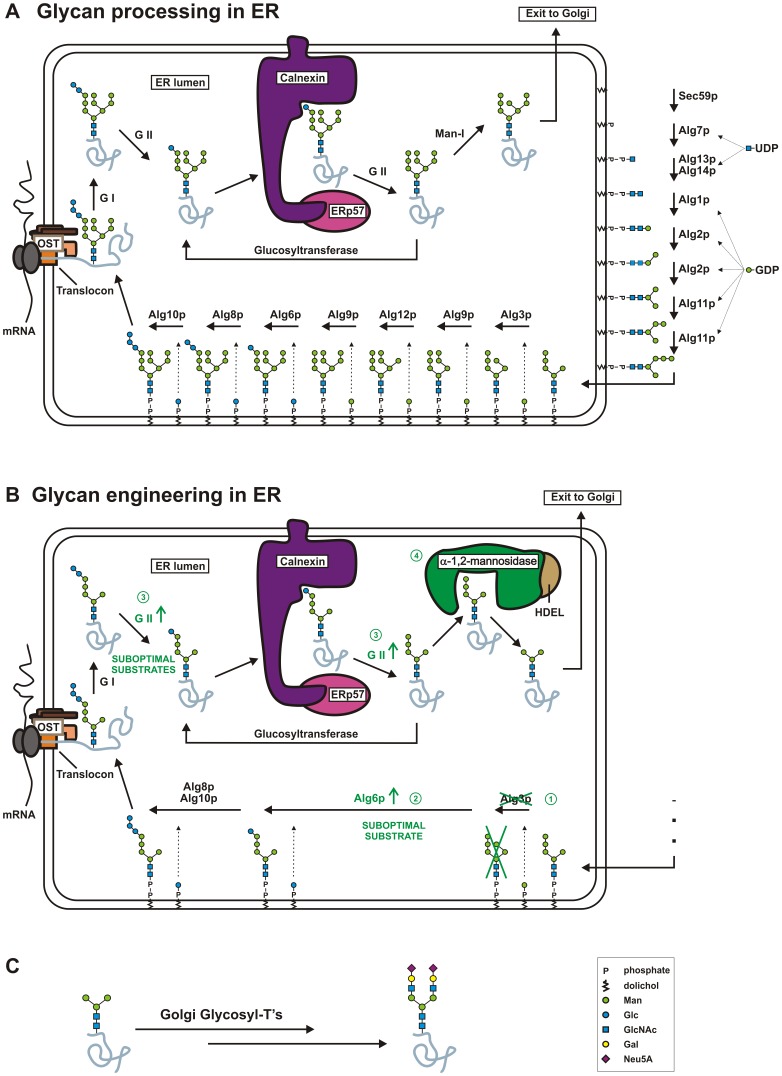
N-glycosylation and engineering thereof in yeast. (A) N-glycosylation in wild type yeast and (B) The approach used to engineer the yeast specific pathway. A: Standard N-glycosylation pathway in the ER. The early steps in N-glycosylation start with the synthesis of a dolichol-linked Man_5_GlcNAc_2_ glycan precursor that flips to the ER lumen, where it is further elongated with mannoses starting with the activity of Alg3p mannosyltransferase. The resulting dolichol-linked Man_9_GlcNAc_2_ precursor is then also glucosylated starting with the activity of Alg6p glucosyltransferase. When complete, the Glc_3_Man_9_GlcNAc_2_ glycan is transferred *en bloc* to the nascent polypeptide chain. These glycans are then subjected to a protein folding quality control process involving de-glucosylation by glucosidases I and II (GI, GII) and re-glucosylation glucosyltransferase. B: The engineering strategies used to obtain a *Y. lipolytica* strain that produces glycoproteins homogeneously modified with the trimannosyl core N-glycan (Man_3_GlcNAc_2_). First, *ALG3* was knocked out (1), then Alg6p was overexpressed (2), then GII was overexpressed (3), and finally α-1,2-mannosidase was overexpressed (4). Conforming to the representation proposed by the Consortium for Functional Glycomics Nomenclature Committee, the green and blue spheres represent mannose (Man) and glucose (Glc), respectively, and blue squares represent N-acetylglucosamine residues (GlcNAc). C: Man_3_GlcNAc_2_-glycans can be further modified to any complex-type N-glycan structure using a combination of glycosyl-transferases, either *in vitro* or *in vivo*.

To generate a *Y. lipolytica* strain producing Man_3_GlcNAc_2_ on its glycoproteins, we engineered the ER-localized components of the N-glycosylation pathway. At the cytoplasmic side of the ER membrane, N-glycosylation starts with the synthesis of a dolichol linked glycan precursor (Figure 1A). The intermediate Man_5_GlcNAc_2_-PP-Dol structure flips to the luminal side of the ER, where it is further elongated, first by the α-1,3-mannosyltransferase Alg3p, and then by other mannosyltransferases until Man_9_GlcNAc_2_ is formed. This dolichol linked sugar is then glucosylated by the α-1,3-glucosyltransferase Alg6p, after which two more glucoses are added. The resultant glycan (Glc_3_Man_9_GlcNAc_2_) is transferred to the nascent polypeptide chain (Figure 1A) [1].

In a process of quality control for protein folding [2], all glucose residues are trimmed sequentially. The first two glucose molecules are removed rapidly by the consecutive action of glucosidase I and II, whereas the last α-1,3-linked glucose residue is removed more slowly by glucosidase II (GII). Monoglucosylated proteins are recognized by calnexin and/or calreticulin. These ER chaperones aid the folding of the glycoprotein and do not reassociate with the glycoprotein once the last glucose residue is removed by GII. If the glycoprotein does not fold properly, it is glucosylated again by the UDP-glucose:glycoprotein glucosyltransferase, after which it again binds calnexin and/or calreticulin and reenters the folding cycle. When the glycoprotein is correctly folded and the sugars are trimmed to Man_8_GlcNAc_2_ by ER mannosidase I, the protein proceeds along the secretory pathway. In the Golgi apparatus of yeasts, the Man_8_GlcNAc_2_ N-glycans are further extended by the addition of mannose and phospho-mannose residues. This elongation is initiated by the α-1,6-mannosyltransferase Och1p [3], [4]. In contrast, higher eukaryotes first trim the glycans to Man_5_GlcNAc_2_ by Golgi mannosidases I and then further modify them to complex type glycans [5]–[7].

Several methods can be envisioned to engineer yeast for the production of homogeneous, universal glycan ‘scaffolds’ on which different types of eukaryotic N-glycans can be built [8]. One approach is to engineer only Golgi-localized processes so that the more essential ER-localized steps of the N-glycosylation pathway are not affected. This has been successfully implemented in *P.* *pastoris* [9], [10]. Another approach is to interfere with the ER steps of the pathway. This is particularly attractive at the *ALG3* step: disruption of *ALG3* is expected to lead to the glycosylation of proteins with Man_5_GlcNAc_2_ N-glycans, which should be easy to trim to Man_3_GlcNAc_2_ with an α-1,2-mannosidase (Figure 1B). Man_3_GlcNAc_2_ is the common core of all types of eukaryotic N-glycans and provides an ideal scaffold for *in vitro* or *in vivo* synthesis of different glycoforms.

However, at least in *S. cerevisiae* [11], [12], the situation is complicated because Man_5_GlcNAc_2_-PP-Dol is glucosylated by Alg6p less efficiently than Man_9_GlcNAc_2_-PP-Dol. Glucosylation of the N-glycan precursor is important for its efficient transfer to nascent proteins by oligosaccharyltransferase, and reduced glucosylation diminishes this transfer. Previous studies have not addressed this shortcoming of this otherwise attractive engineering approach. Here, we report that the glucose residues on glycoproteins produced in *alg3* strains are not removed efficiently by *Yarrowia* GII, and we describe the engineering strategy we used to solve this problem (Figure 1B). Through this integrated ‘systems engineering’ approach, we succeeded in creating a glyco-engineered *Y. lipolytica* strain that produces glycoproteins homogeneously modified with the trimannosyl core N-glycan (Man_3_GlcNAc_2_).

## Results

### *ALG3* Gene Knock-out

In order to alter *Y. lipolytica* to produce heterologous proteins glycosylated with Man_3_GlcNAc_2_, we interfered with biosynthesis of the core N-glycan (Figure 1B, step1). Elimination of Alg3p α-1,3-mannosyltransferase prevents the addition of an α-1,3-mannose to the α-1,6-arm of the ER Man_5_-PP-Dol structure. Knock-out of *ALG3* should lead to accumulation of its substrate, Man_5_GlcNAc_2_ [13], [14].

**Figure 2:**
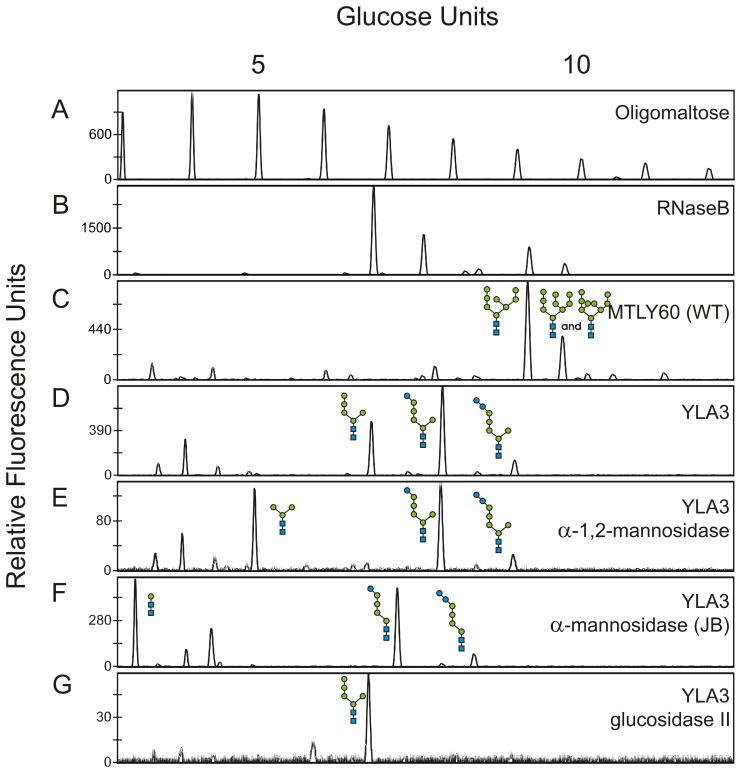
Identification of N-glycans by exoglycosidase digestion and DSA-FACE analysis. A: Oligomaltose reference. B, N-glycans from RNaseB reference. C–G, N-glycans from different strains: C, MTLY60 wild type strain; D, *alg3* knock-out strain; E, The same as D but treated with α-1,2-mannosidase; F, The same as D but treated with JB α-mannosidase; G, The same as D but treated with glucosidase II. The N-glycan structures in the *alg3* knock-out strain are consistent with Man_5_GlcNAc_2_, GlcMan_5_GlcNAc_2_ and Glc_2_Man_5_GlcNAc_2_.

To disrupt the *Y. lipolytica ALG3* gene, we constructed a plasmid that includes parts of the promoter and terminator of *ALG3* and has a *URA3* selection marker cassette (pYLalg3PUT). The *Not*I and *Pac*I sites were used to linearize the vector in order to remove the *E. coli* related DNA elements before transformation of wild type (WT) *Y. lipolytica* MTLY60 (Table 1). Double homologous recombination at the promoter and terminator sites replaced *ALG3* with the *URA3* selectable marker, which resulted in the *alg3*::URA3 mutant strain YLA3 (Table 1). To study the effect of this mutation, we analyzed the N-glycan profile of proteins that completely traverse the yeast’s secretory system, *i.e*. cell wall mannoproteins. Whereas the wild type mannoproteins contained mainly Man_8_GlcNAc_2_ and Man_9_GlcNAc_2_ N-glycans (Figure 2, panel C), the *alg3* mutants proteins had three glycan structures (Figure 2, panel D). As expected, one of these structures ran at about the same position in electrophoresis as the Man_5_GlcNAc_2_ sugar structure of RNaseB, but two others ran at positions corresponding to one and two extra monosaccharide units (Figure 2, panel D). This was the case for all transformants that were confirmed by PCR on gDNA to be *alg3* knock-outs.

**Table 1:** *Y. lipolytica* strains used in this study.

*Y.l.* strains	Genotype	Reference
MTLY60	MatA ura3-302 leu2-270 xpr2-322_lip2_lip7_lip8	Fickers *et al.*, 2005
YLA3	MTLY60 with *alg3*::URA3	This work
YLA3–A6	MTLY60 with *alg3*::ALG6-URA3	This work
YLTBGIIA	As YLA3-A6+overexpr of *Tb* GII α	This work
YLTBGIIAHDEL	As YLA3-A6+overexpr of *Tb* GII αHDEL	This work
YLTBpreGIIAHDEL	As YLA3-A6+overexpr of LIP2pre *Tb* GII α HDEL	This work
YLYLGIIA	As YLA3-A6+overexpr of *Yl* GII α	This work
YLYLGIIAHDEL	As YLA3-A6+overexpr of *Yl* GII αHDEL	This work
YLYLGIIAB	As YLA3-A6+overexpr of *Yl* GII α, βα, β	This work
YLANGIIA	As YLA3-A6+overexpr of *An* GII α	This work
YLANGIIAB	As YLA3-A6+overexpr of *An* GII α,β	This work
YLMAN	As YLANGIIAB+overexpr of α-1,2-mannosidase	This work

To further elucidate the structures of the two additional N-glycans, we performed exoglycosidase digests with α-1,2-mannosidase, Jack Bean (JB) α-mannosidase and purified rat liver GII and analysed the products using capillary electrophoresis. The peak that had reached the same position as Man_5_GlcNAc_2_ of the RNaseB marker shifted two glucose units after α-1,2-mannosidase treatment (Figure 2, panel E) and four glucose units after broad-specificity α-mannosidase (JB) digestion (Figure 2, panel F). This fits with the dolichol-linked Man_5_GlcNAc_2_ structure, as expected. The additional two glycans are not affected by α-1,2-mannosidase digestion. Also, both peaks shifted only one glucose unit upon α-mannosidase (JB) digestion. However, both glycans were sensitive to GII digestion and were converted to Man_5_GlcNAc_2_ (Figure 2, panel G). In the light of what is known about the canonical eukaryotic N-glycosylation pathway, these findings are consistent with the three observed N-glycan structures in the *alg3* mutant being Man_5_GlcNAc_2_, Glcα1,3Man_5_GlcNAc_2_ and Glcα1,3Glcα1,3Man_5_GlcNAc_2_ (Figure 2, panel D).

### Compensation for Underoccupancy of the N-glycan Sites by Overexpressing *ALG6*

**Figure 3:**
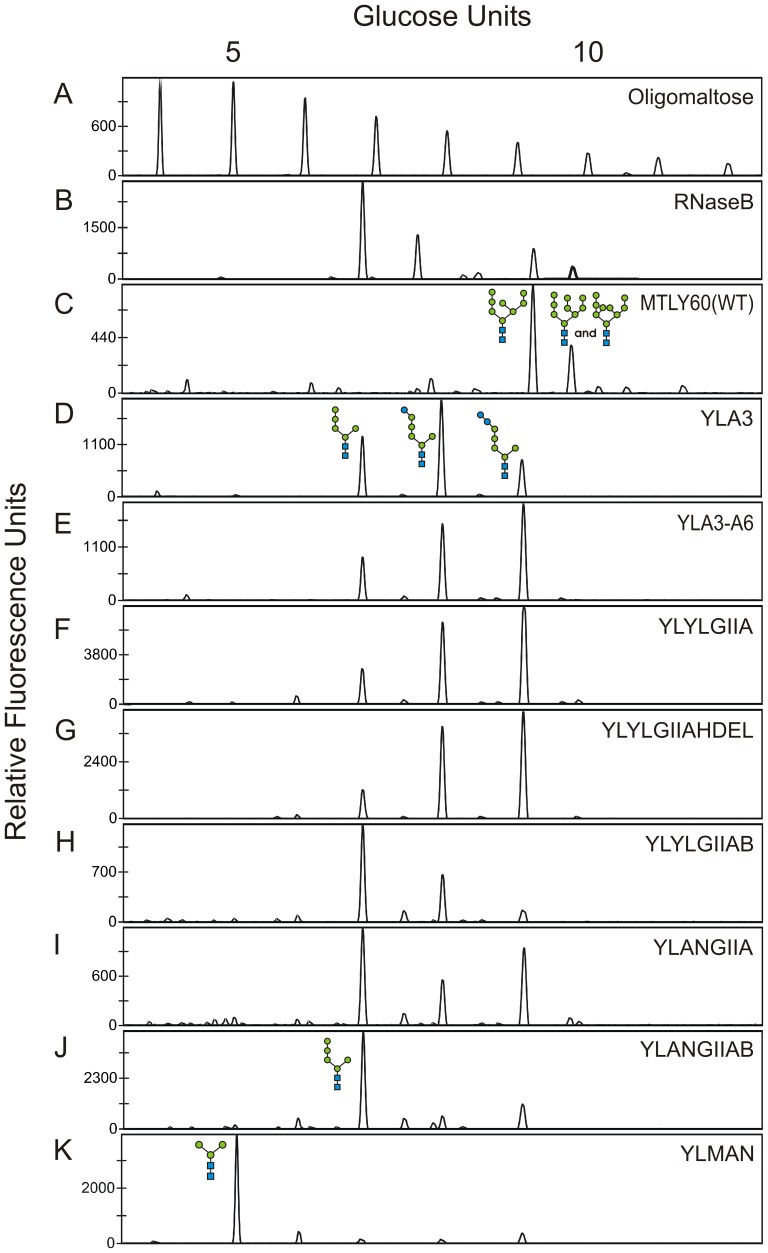
DSA-FACE analysis of engineered *Y. lipolytica* strains. A, oligomaltose reference. B–K, N-glycans derived from different sources: B, bovine RNaseB reference; C, MTLY60 wild type strain; D, *alg3* knock-out strain; E, *alg3* mutant strain overexpressing Alg6p. F–J, the *alg3* mutant strain overexpressing Alg6p engineered with: F, *Y. lipolytica* GIIα; G, *Y. lipolytica* GIIα HDEL-tagged; H, both α and β subunits of *Y. lipolytica* GII; I, the HDEL-tagged *A. niger* GIIα; J, both α and β subunits of *A. niger* GII. K, The latter strain engineered with an HDEL-tagged *T. reesei* α-1,2-mannosidase. This fully engineered strain produces glycoproteins with more than 85% trimannosyl core N-glycans.

The *alg3* mutation in *S. cerevisiae* causes underoccupancy of N-glycosylation sites [12], [13], [15]–[17]. Efficient transfer of the dolichol linked N-glycan precursor to a protein by the oligosaccharyltransferase complex (OST) requires the triglucosyl glycotope on the dolichol-linked precursor [1], [18]. The first glucosyltransferase, Alg6p, can glucosylate the Man_5_GlcNAc_2_-PP-Dol structure in *alg3 S. cerevisiae* [12], but with low efficiency. This results in underglucosylation of the dolichol linked precursor, poor transfer by OST, and reduced occupancy of N-glycosylation sites. Anticipating this problem, we incorporated an Alg6p constitutive overexpression cassette in the *alg3* knock-out vector (Figure 1B, step2). The resultant vector (pYLalg3PUT-ALG6) was transformed into WT *Y. lipolytica* MTLY60, yielding strain YLA3–A6 (Table 1). Upon DSA-FACE analysis of the N-glycans derived from mannoproteins, all transformants in which *alg3* knock-out was confirmed by PCR exhibited a change in glycosylation pattern. The proportion of glucosylated Man_5_GlcNAc_2_ increased substantially (Figure 3, panel E) compared to the *alg3* mutant without Alg6p overexpression (Figure 3, panel D). This indicates that Alg6p activity was indeed augmented and clearly shows that the endogenous *Y. lipolytica* GII activity was insufficient to deglucosylate its suboptimal Glc_1-2_Man_5_GlcNAc_2_ substrates.

**Figure 4:**
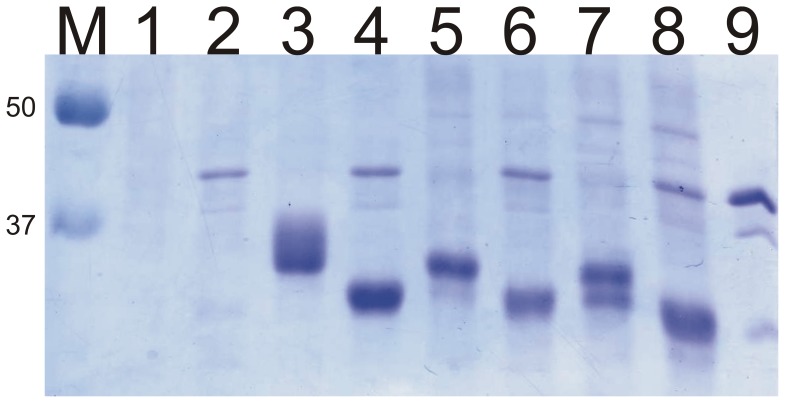
SDS-PAGE evaluation of underoccupancy of N-glycan sites in lipase 2 after inactivation of *alg3*. 1, Wild-type strain (WT, MTLY60). 3, The same as lane 1 but overexpressing lipase2. 5, The alg3 knock-out strain overexpressing lipase 2 and Alg6p. 7, The alg3 knock-out strain overexpressing lipase2. Lanes 2, 4, 6 and 8, the same as 1, 3, 5, and 7, respectively, but treated with PNGaseF. A hyperglycosylation smear is observed when lipase2 is overexpressed in the WT strain. For the alg3 mutant strain expressing lipase2, two distinct bands are visible, which is consistent with site underoccupancy largely compensated for by Alg6p overexpression. Lane 9: PNGaseF preparation used for the digestions shown in Lane 2, 4, 6 and 8.

To evaluate the underoccupancy of N-glycosylation sites in our different strains, we examined the N-glycosylation of overexpressed *Y. lipolytica* lipase 2 (LIP2), which has two glycosylation sites [19], [20]. We analyzed the pattern of secreted proteins before and after N-deglycosylation with PNGaseF. For the wild type strain, a single LIP2 band with a smear of hyper-N-glycosylation is observed (Figure 4, lane 3). In the *alg3* knock-out strain, LIP2 is found in two bands (Figure 4, lane 7), the top one at the same MW as the non-hyperglycosylated wild type-produced protein, and the bottom one at an intermediate position between the wild type-produced protein and the fully de-N-glycosylated protein. The bottom band is much less abundant in the preparation from the *alg3* mutant strain overexpressing Alg6p (Figure 4, lane 5). The bands are separated by 1–2 kDa and they collapse into one band when the N-glycans are removed by PNGaseF digestion (Figure 4, lane 4, 6 and 8). These results indicate that the N-glycosylation sites are underoccupied in the *alg3* mutant. As intended, overexpression of Alg6p largely compensated for this underoccupancy, because only one band is visible on the protein gel (Figure 4, lane 5). It should be noted that this phenotype was observed in cells in mid-log phase of growth, and that it was much less pronounced in stationary-phase cells (data not shown). The difference is probably due to the considerably slower flux of proteins through the N-glycosylation pathway in stationary phase.

Interestingly, no hyperglycosylation of LIP2 was seen in the *alg3* and *alg3ALG6* strains, which means that our strategy need not involve knocking out any Golgi mannosyltransferases to obtain homogeneous glycosylation, contrary to previous approaches [9], [10].

Consequently, we solved the underglycosylation problem of the *alg3* mutant by overexpressing Alg6p, but this was at the expense of further augmenting the fraction of undesired glucosylated Man_5_GlcNAc_2_ derivatives.

### Removal of Capping Glucoses

In strains in which *alg3* is disrupted, the N-glycans are capped by GII-hydrolyzable glucose residues. This type of capping is more pronounced when the *ALG6* gene is overexpressed. Since the presence of these glucose residues prevents conversion of Man_5_GlcNAc_2_ to Man_3_GlcNAc_2_ by an introduced α-1,2-mannosidase (Figure 1B, step 4), our next objective was to eliminate those glucose residues by further *in vivo* engineering.

#### Removal of capping glucose residues: mutanase and *T. brucei* GII

**Figure 5:**
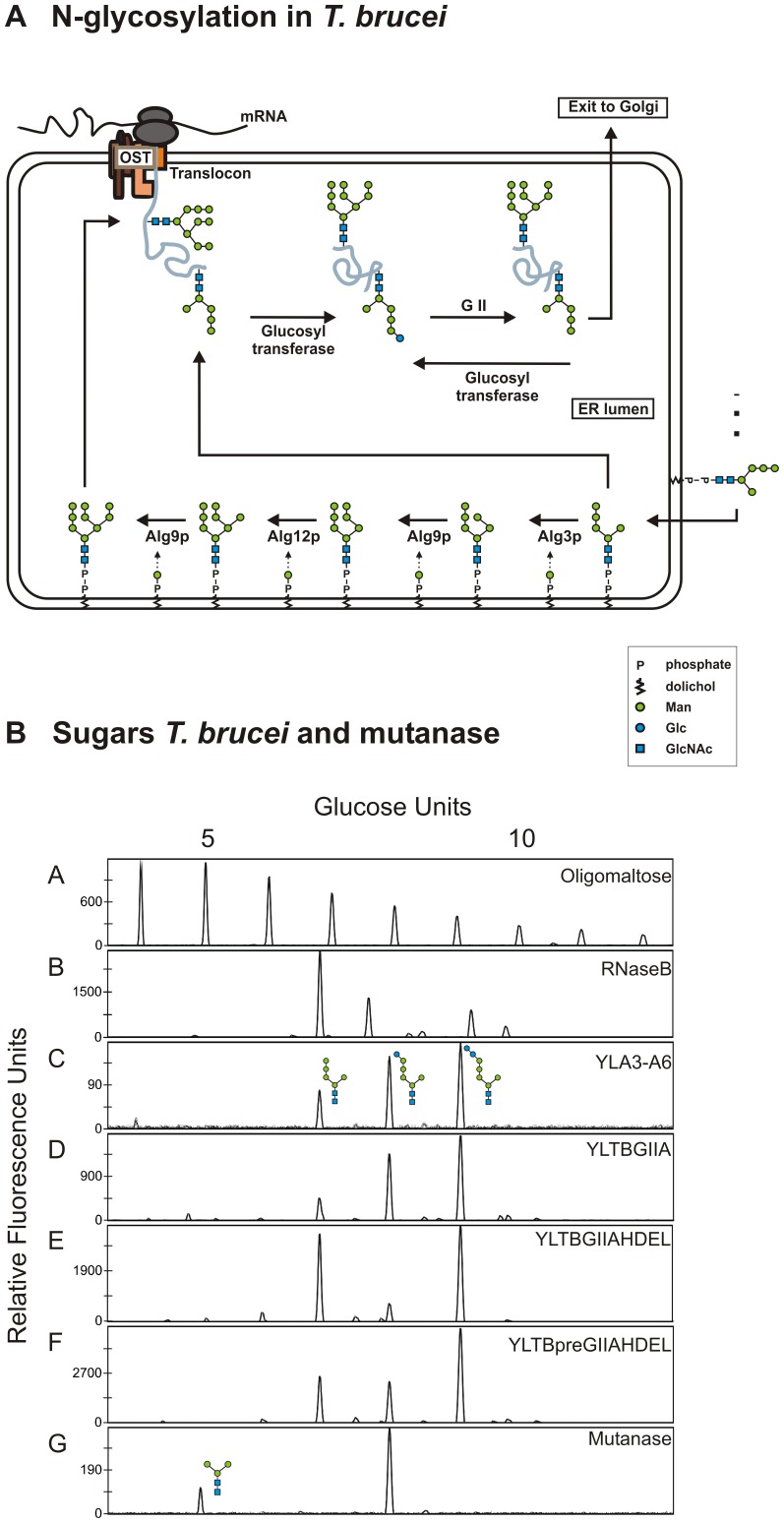
*T. brucei* GII and mutanase tested as engineering approach. (**A**) **The dual N-glycosylation system in** ***T. brucei*** **.** Both Man_9_GlcNAc_2_ and Man_5_GlcNAc_2_ can be transferred to proteins. Next, these proteins are reglucosylated and deglucosylated in the folding cycle by glucosyltransferase and GII, respectively. (**B**) **DSA-FACE analysis of reference N-glycans and N-glycans derived from strains engineered with** ***T. brucei*** **GII or treated with mutanase.** A, Oligomaltose reference. B, N-glycans from RNaseB reference. C, N-glycans from the *alg3* mutant strain overexpressing Alg6p. D-F, N-glycan from the *alg3* mutant strain overexpressing Alg6p and engineered in different ways: D, engineered with *T. brucei* GII; E, engineered with *T. brucei* GII with HDEL tag; F, engineered with *T. brucei* GII with HDEL tag and pre-lip2 signal. G, N-glycans derived from the *alg3* mutant strain overexpressing Alg6p treated with mutanase.

We examined the possibility of using the mutanase of *Trichoderma harzianum* to remove the capping glucose residues on the Man_5_GlcNAc_2_ glycans. Both unwanted glucose residues are α-1,3-linked to the rest of the sugar, and this mutanase has α-1,3-glucosidase activity. A dilution series of the Novozyme 234 mutanase preparation was added to the oligosaccharides derived from the YLA3-A6 strain (Man_5_GlcNAc_2_, GlcMan_5_GlcNAc_2_ and Glc_2_Man_5_GlcNAc_2_). The DSA-FACE profile (Figure 5B, panel G) shows that Glc_2_Man_5_GlcNAc_2_ was effectively hydrolyzed to GlcMan_5_GlcNAc_2_. However, GlcMan_5_GlcNAc_2_ was not deglucosylated further. It should be noted that Man_5_GlcNAc_2_ was also trimmed, most probably by a contaminating mannosidase in the crude enzyme mixture. Since complete deglucosylation could not be obtained with this mutanase, we abandoned this approach.

As an alternative strategy, we overexpressed the *T. brucei* GII α-subunit. *T. brucei* uses a dual N-glycosylation system that can transfer both Man_9_GlcNAc_2_ and Man_5_GlcNAc_2_ to proteins (Figure 5A) [21]. Furthermore, unlike organisms that exclusively transfer Glc_3_Man_9_GlcNAc_2_, the GII enzyme in *T. brucei* uses GlcMan_5_GlcNAc_2_ as a preferred substrate [22]. Therefore, we tested whether the *T. brucei* enzyme can deglucosylate these structures in our engineered strains. We transformed the YLA3–A6 strain with pYLHmAXTbGIIa, which resulted in a YLTBGIIA strain (Table 1) and analyzed its cell wall mannoprotein glycans. No deglucosylation was observed (Figure 5B, panel D). As GII is heterodimeric [23], we considered the possibility that the α-subunit of *T. brucei* GII cannot dimerize with the β-subunit of *Y.* *lipolytica* GII and would thus not be retained in the endoplasmic reticulum. So we introduced an HDEL ER-retrieval tag at the C-terminus of the α-subunit of *T. brucei* GII. Moreover, we expressed the *T. brucei* enzyme once with its own signal peptide and once with the *Y. lipolytica* LIP2 signal peptide in the YLA3–A6 strain (yielding strains YLTBGIIAHDEL and YLTBpreGIIAHDEL, respectively) (Table 1). N-glycan analysis of the clones overexpressing the HDEL-tagged α-subunit showed reduced abundance of the mono-glucosylated Man_5_GlcNAc_2_ peak (Figure 5B, panel E and F), whereas the di-glucosylated Man_5_GlcNAc_2_ structure was not hydrolyzed. Evidently, this latter structure is not a substrate for the *T. brucei* GII. Consequently, this engineering approach also did not solve our problem, so we abandoned it.

#### Removal of capping glucoses by overexpression of the endogenous GII

To eliminate mono- and di-glucosylated Man_5_GlcNAc_2_ structures *in vivo*, the YLA3–A6 strain was genetically engineered to overexpress the *Y. lipolytica* GII. This enzyme is a heterodimer consisting of two subunits, of which the α-subunit is catalytically active [23] and contains a GH31 family domain [24]. We started by overexpressing the α-subunit in our YLA3–A6 strain. Glucosylation of the various glycans in the resultant strain, YLYLGIIA, was not reduced (Figure 3, panel F *versus* panel E).

It is believed that the β-subunit, which contains an HDEL tag, serves primarily to retain the α-subunit in the ER [23], [25]–[27]. Therefore, first we tried mimicking the β-subunit’s function by adding an HDEL tag to the C-terminus of the α-subunit of the *Y.* *lipolytica* GII. This way, the tag would serve to retrieve the enzyme from the Golgi apparatus to the ER via COPI vesicles and thereby help to maintain the enzyme at its site of action. Again, α-glucose removal was not improved in any of the transformation clones of the resultant YLYLGIIAHDEL strain (Figure 3, panel G).

Several studies have indicated the necessity of the β-subunit of the GII complex for maturation, solubility, stability and enzymatic activity on natural substrates [25]–[29]. Overexpression of the α subunit of *Y. lipolytica* GII alone was not sufficient to reduce the unwanted glucosylation on the Man_5_GlcNAc_2_ glycan. Therefore we simultaneously overexpressed the β-subunit in two strains that overexpress the *Y. lipolytica* GII α-subunit with or without HDEL tag and we tested both the hp4d and the TEF promoter. We retained the clone with the best glycan profile, *i.e*. the one that removed α-glucose most efficiently. The best result was obtained in a strain that overexpressed the *Y. lipolytica* GII α-subunit with the HDEL tag, with a slightly improved effect when the *Y. lipolytica* GII β-subunit was expressed from the TEF promoter compared to the hp4d promoter. Therefore, we created a strain that overexpressed both the *Y. lipolytica* GII α and β-subunit driven by the TEF promoter. The strain was named YLYLGIIAB (Figure 3, panel H). However, though overexpression of both α- and β-subunits of *Y. lipolytica* GII significantly reduced the proportion of glucosylated Man_5_GlcNAc_2_, it was still insufficiently effective for homogeneous glycoprotein production.

#### Removal of capping glucoses by overexpression of the *A. niger* GII

Kainz and colleagues [30] recently reported that knockout of *ALGC*, the *ALG3* homologue in the filamentous fungus *A. niger,* leads to the synthesis of Man_3-6_GlcNAc_2_ glycans. *In vitro* digestion of these glycans with α-1,2-mannosidase gave almost exclusively Man_3_GlcNAc_2_ [30]. Hence, no glucosylated glycan structures were detected when the *ALG3* gene was disrupted in *A. niger*. Therefore, we assumed that the GII of *A. niger* can cope better with the alterations in N-glycan substrate structures caused by inactivation of the ER-mannosyltransferase Alg3p. Indeed, overexpression of the HDEL-tagged α-subunit of *A. niger* GII alone in our *Y. lipolytica alg3* strain overexpressing ALG6, *i.e*. YLA3–A6, resulted in trimming of the glucosylated Man_5_GlcNAc_2_ forms in the newly made YLANGIIA strain (Figure 3, panel I). No differences were seen between the strains that overexpressed the α-subunit of *A. niger* GII under control of the TEF or under control of the hp4d promoter (data not shown). We subsequently overexpressed the β-subunit of *A. niger* GII in the YLANGIIA strain that overexpressed the HDEL-tagged α-subunit of *A. niger* GII, also under control of the TEF promoter. The resultant strain was named YLANGIIAB. Analysis of the glycan structures on glycoproteins produced by this strain showed very efficient conversion of glucosylated to non-glucosylated Man_5_GlcNAc_2_ glycan structures (Figure 3, panel J), which represented about 80% of the total cell wall mannoprotein N-glycan pool.

### Overexpression of ER-targeted α-1,2-mannosidase Leads to Production of Man_3_GlcNAc_2_

As a final step in our N-glycan engineering (Figure 1B, step 4), we aimed at converting Man_5_GlcNAc_2_ to core Man_3_GlcNAc_2_ glycan structures. Therefore, we overexpressed a *Y. lipolytica*-optimized ER-targeted *T. reesei* α-1,2-mannosidase [31], [32] in the *alg3* knock-out strain overexpressing Alg6p and the *A. niger* GIIα/β, *i.e*. YLANGIIAB. The resulting strain, YLMAN, produces homogeneous Man_3_GlcNAc_2_ (>85%) (Figure 3, panel K).

## Discussion

*Y. lipolytica* has emerged as a suitable system for heterologous protein expression [33]. With the increasing importance of yeasts as an alternative host for recombinant protein production, it has become important to glyco-engineer yeasts for production of humanized glycans for therapeutic purposes. We aimed to engineer the *Yarrowia* ER glycosylation pathway for the production of the Man_3_GlcNAc_2_ core N-glycan structure, which can be converted to any desired mammalian N-glycan using Golgi glycosyltransferases (Figure 1C).

Upon disruption of the *ALG3* gene in *Y. lipolytica*, we observed the expected Man_5_GlcNAc_2_ (dolichol-linked type) as well as two additional glycan structures: GlcMan_5_GlcNAc_2_ and Glc_2_Man_5_GlcNAc_2_. Both glucose residues could be removed *in vitro* by purified rat liver GII. It has also been reported that N-glycosylation sites of secretory proteins are underoccupied in *alg3* mutants [12], [13], [15]–[17]. Various studies have shown that the glucose residues on the lipid-linked oligosaccharide facilitate the transfer of the oligosaccharide to protein [1], [18]. Nonglucosylated or partially glucosylated oligosaccharides can be transferred to protein, but with a reduced efficiency. In *alg3* mutants of baker’s yeast, the resulting Man_5_GlcNAc_2_ lipid-linked glycan is not glucosylated efficiently [12]. Apparently, the 6′ branch of the oligosaccharide is a major structural determinant in the specificity and activity of the Alg6p, dolichol-P-Glc:Man9GlcNAc2-PP-Dol glucosyl transferase, which is the first glucosyltransferase in the ER [16], [34]. We anticipated this problem and avoided it by constitutively overexpressing the *Y. lipolytica ALG6* gene. Indeed, overexpression of *ALG6* largely remedied the defect in N-glycosylation site occupancy in the lipase secreted by the *alg3* mutant. However, this complemented strain secreted proteins with more Man_5_GlcNAc_2_ glucosylation, most likely because of the transfer of a larger fraction of nonglucosylated Man_5_GlcNAc_2_ to proteins.

Remarkably and beneficially, *Y. lipolytica* Golgi glycosyltransferases does not seem to further modify the glycans upon disruption of the *ALG3* gene. This was also reflected in the increased homogeneity of secreted LIP2 lipase on SDS-PAGE gels. Most likely, *Yl*Och1p does not recognize the ER-type Man_5_GlcNAc_2_ or its glucosylated derivates.

In contrast, N-glycans released from an *alg3och1* mutant strain of *P. pastoris* contain the expected Hex_5_GlcNAc_2_ structure, as well as large quantities of glycans of higher molecular weight ranging from Hex_6_GlcNAc_2_ to Hex_12_GlcNAc_2_ [35]. Upon treatment with α-1,2-mannosidase, the Man_5_GlcNAc_2_ was converted to Man_3_GlcNAc_2_, which is consistent with the *alg3* Man5 structure. The other glycans, however, were mostly resistant to treatment with broad-specificity α-mannosidase. Amongst these, only Hex_6_GlcNAc_2_ was shown to contain glucose, which is consistent with a GlcMan_5_GlcNAc_2_ structure [35]. The presence of larger structures implies the existence of *P. pastoris* Golgi glycosyltransferases capable of acting on these substantially truncated substrates. This is clearly different from the situation in *Yarrowia*. In a *S. cerevisiae alg3sec18* mutant, a substantial proportion of the glycan chains on the model protein invertase were the mono-, di- and triglucosylated Man_5_GlcNAc_2_ structures [12], [16], [36].

In contrast, in the plant *Arabidopsis thaliana*, an *alg3cgl* mutant yielded Man_3-4_GlcNAc_2_ glycans, which led to the hypothesis that an aberrant Man_5_GlcNAc_2_ structure, once it is transferred to a protein, is trimmed by the Golgi α-1,2-mannosidase [37]. Similarly, analysis of whole cell extracts from the filamentous fungus *A. niger algC* knock-out (the *ALG3* homologue) revealed the presence of Man_3-6_GlcNAc_2_ N-glycans [30]. Moreover, proteins secreted by an *alg3* mutant of the yeast *Hansenula polymorpha* contain almost no glucosylated glycans [38]: model glycoproteins contain predominantly Man_5_GlcNAc_2_. The less abundant Hex_6-8_GlcNAc_2_ structures can be almost completely converted to Man_3_GlcNAc_2_ by *in vitro* digests with α-1,2- and α-1,6-mannosidases. Deletion of the endogenous *OCH1* gene encoding the initiating α-1,6-mannosyltransferase decreases the overall abundance of Hex_6-8_GlcNAc_2_ structures and only a minor fraction of Hex_6_GlcNAc_2_ remains. This Hex_6_GlcNAc_2_ glycan quite likely contains a capping glucose residue [38].

The presence of glucose residues on the *alg3* Man_5_GlcNAc_2_ glycans implies either the existence of an endogenous glucosyltransferase or, more likely, insufficient activity of ER-resident GII, which normally cleaves both α1,3-linked glucose residues successively from Glc_2_Man_9_GlcNAc_2_. GII’s substrate specificity includes the 6′ pentamannosyl branch of its glucose-containing oligosaccharide substrates. Its activity seems to decrease with reduction of the number of mannoses on the 6′ branch of the N-glycan substrate. Mammalian GII activity was several times higher with Glc_1-2_Man_9_GlcNAc_2_ as substrate than with Glc_1-2_Man_7_GlcNAc_2_. Moreover, oligosaccharides lacking the four outermost mannose residues on the 6′ branch were very poor substrates [39]. Similar results were obtained by other investigators [40]–[43]. More recently, it was found that the rate of GII-mediated trimming is specifically dependent on the presence of the α-1,2-linked mannose on the C-arm [44]. The β-subunit of GII contains a mannose-6-phosphate-homology (MRH) domain that recognizes carbohydrates and contributes to substrate recognition [45]. Sequence alignments indicated that all residues involved in mannose binding in the MRH domain are conserved in GII β, except for those that interact with the phosphate group. Indeed, there is evidence that the GII β-subunit plays a key role in enhancing the specific activity of the heterodimeric GII enzyme towards natural N-glycan substrates [28], [29], [46]–[49].

From all the above observations, it can be concluded that, GII of *Y. lipolytica* is much more specific for its natural substrate than, for example, the GII of *A. thaliana* or *A. niger*. Here, we used this broader substrate specificity of *A. niger* GII to reduce the glucosylation of our YLA3–A6 strain.

The feasibility of our integrated system’s engineering approach illustrates the current level of understanding of the N-glycosylation pathway’s intricacies. We anticipate that this strain will find use in the structure-function analysis of N-glycan modifications in many settings, such as in the fine-tuning of biopharmaceutical protein N-glycans to particular therapeutic goals.

## Materials and Methods

### Strains, Reagents and Culture Conditions

*Escherichia coli* strains MC1061, TOP10, and DH5α were used for the amplification of recombinant plasmid DNA.

*Yarrowia lipolytica* MTLY60 (Table 1) [50] was used as parent strain. All yeast strains were cultured at 28°C. They were grown on YPD (20 g/L dextrose, 20 g/L bacto-peptone and 10 g/L yeast extract) or MM (1.7 g/L yeast nitrogen base (YNB) without amino acids and ammonium sulfate, 10 g/L glucose, 5 g/L NH_4_Cl, 50 mM K^+^/Na^+^ phosphate buffer pH 6.8, and 7.7 g/L Complex Serum-free Medium (CSM)); for selection of Ura^+^ and Leu^+^ transformants, 7.7 g/L CSM –ura or CSM –leu was added instead of CSM.

### Standard Genetic Techniques

For transformation of *Y. lipolytica*, competent cells were prepared as described [51]. Briefly, cells were pretreated with lithium acetate and incubated with the DNA to be transformed together with salmon sperm carrier DNA. PEG 4000 was added, and after a heat shock at 42°C, cells are plated on selective plates.

Genomic DNA was isolated using the MasterPure™ Yeast DNA Purification Kit according to the instructions of the manufacturer (Epicenter Biotechnologies). PCR amplification was performed in a volume of 50 µL containing 20 mM Tris-HCl pH 8.4, 50 mM KCl, different concentrations of MgCl_2_, 0.4 mM of dNTPs, 50 ng of template DNA, 50 pmol of primers, and 2.5 units of either *Taq* or *Pfu* DNA polymerase. Cycling conditions were as follows: denaturation at 94°C for 10 min followed by hot start at 80°C and 30 cycles of 94°C for 45 s, suitable annealing temperature for 45 s, and extension at 72°C for 1 min per kbp, followed by 10 min of final extension at 72°C.

DNA fragments in PCR reactions and those recovered from gels were purified using NucleoSpin extract II (Macherey-Nagel).

### Vector Construction

#### Knocking out the *ALG3* gene

We used a knock-out strategy that makes use of the Cre-lox recombination system, which facilitates efficient marker rescue [52]. The genomic region upstream of the *ALG3* ORF (GenBank Accession No: XM_503488; Genolevures: YALI0E3190g) was amplified from genomic DNA of *Y. lipolytica* MTLY60 by PCR with primers ALG3Pfw and ALG3Prv (Table 2) using *Taq* polymerase (Invitrogen, Carlsbad, CA, USA). The overhanging A was removed with T4 DNA polymerase (Fermentas, Burlington, Ontario, Canada). The genomic region downstream of the *ALG3* ORF was amplified from genomic DNA of *Y. lipolytica* MTLY60 by PCR with primers ALG3Tfw and ALG3Trv (Table 2) using *Pfu* DNA polymerase (Fermentas). The presence of overlapping primer sequences containing I*-Sce*I restriction sites allowed the linking of the fragments by PCR with primers ALG3Pfw and ALG3Trv using *Taq* polymerase. This co-amplicon was then subcloned in pCR-2.1-TOPO-TA (Invitrogen, Carlsbad, CA, USA) and sequenced. It was then cloned between the *Not*I and *Pac*I sites in a derivative of pBluescriptIISK (Stratagene, Cedar Creek, Texas, USA) to yield pBLUYLalg3PT. Next, the *URA3* selection marker flanked by *lox* sites originating from pKS-LPR-URA3 [52] (a gift from J.M. Nicaud, INRA) was inserted in the introduced I*-Sce*I site between upstream and downstream regions, yielding pYlalg3PUT. Similarly, pYlalg3PLT was constructed by exchanging the *URA3* cassette in pYlalg3PUT with the *LEU2* selection marker from pKS-LPR-LEU2 [52] by means of I-*Sce*I digestion.

**Table 2:** Primers used in this study.

Primer name	Sequence (5′→3′)	Restriction site
ALG3Pfw	CAGTGCGGCCGCACTCCCTCTTTTCACTCACTATTG	*Not*I
ALG3Prv	CATTACCCTGTTATCCCTACGCTCAGATCCAATTGTTTTGGTGGTC	I-*Sce*I
ALG3Tfw	GTAGGGATAACAGGGTAATGCTCTCAAGGACGGACCAGATGAGACTGTTATCG	I-*Sce*I
ALG3Trv	GACTTTAATTAAACCCTATGTGGCACCTCAACCCACATCTCCCGTC	*Pac*I
ALG6fw	CAGTGGATCCATGAACTCTCCTATTTTCACTACCG	*Bam*HI
ALG6rv	GACTCCTAGGAAGCTTCCAGGTTACAAGTTGTTAC	*Avr*II
YlGlucIIαfw	GTCCAGATCTATGAAAACGACGTTAGTAGCGCTGC	*Bgl*II
YlGlucIIαrv	CTAGCCTAGGTTAAGAGAAGGACATGGCCCAAG	*Avr*II
TbGlucIIαfw	GTCCGGATCCATGCTATCGCTTGTGCTATCGTTG	*Bam*HI
TbGlucIIαrv	CTAGCCTAGGCTATCTCTTCAGCACAATGGTCC	*Avr*II
YlGlucIIαHDELrv	CTAGCCTAGGTTACAACTCGTCGTGAGAGAAGGACATGGCCCAAG	*Avr*II
TbGlucIIαHDELrv	CTAGCCTAGGCTACAACTCGTCGTGTCTCTTCAGCACAATGGTCC	*Avr*II
YlGlucIIβfw	GTCCGGATCCATGAAAATCTCGGCTATCTTCG	*Bam*HI
YlGlucIIβrv	CTAGCCTAGGCCTACAGCTCATCATGTTTTCC	*Avr*II
LIP2prefw	GATCC ATGAAGCTTTCCACCATCCTCTTCACAGCCTGCGCTACCCTGGCCGCGGTAC	*‘Bam*HI’
LIP2prerv	CTAGG TACCGCGGCCAGGGTAGCGCAGGCTGTGAAGAGGATGGTGGAAAGCTTCATG	*‘Avr*II’

Restriction sites in ‘’ refer to overhanging parts of them.

#### Cloning the *ALG6* gene

The ORF (1725 bp) of *ALG6* together with the 415-bp downstream region (GenBank Accession No: XM_502922; Genolevures: YALI0D17028g) was cloned from genomic DNA of *Y. lipolytica* MTLY60 by PCR with primers ALG6fw and ALG6rv (Table 2) using *Pfu* DNA polymerase. The amplified fragment was cloned in pCR-Blunt-II-TOPO (Invitrogen, Carlsbad, CA, USA) and sequenced. Next, it was cloned between the *Bam*HI and *Avr*II sites of pYLHmA (pINA1291) [53], which contains the hp4d promoter [54] and the LIP2 terminator. It was then subcloned in the intermediate vector pBLUYLalg3PT in the unique *Cla*I and *Hind*III restriction sites present in the downstream region of *ALG3*. The *URA3* selection marker flanked by *lox* sites, which was obtained from pKS-LPR-URA3, was inserted in the introduced I-*Sce*I site between promoter and terminator fragments of the *ALG3* gene. The resultant plasmid was named pYlalg3PUT-ALG6. Similarly, pYlalg3PLT-ALG6 was made by exchanging the *URA3* cassette in pYlalg3PUT-ALG6 with the *LEU2* selection marker from pKS-LPR-LEU2 be means of I-*Sce*I digestion.

#### Cloning the GII alpha-subunit of *Y. lipolytica* with and without HDEL tag

The ORF (2766 bp) of the *Y. lipolytica* GII α-subunit gene (GenBank Accession No: XM_500574) was amplified from genomic DNA of *Y. lipolytica* MTLY60 by PCR with primers YlGlucIIαfw and YlGlucIIαrv (Table 2) using *Pfu* DNA polymerase. The PCR fragment was cloned in pCR-Blunt-II-TOPO (Invitrogen, Carlsbad, CA, USA) and confirmed by Sanger sequencing. Next, it was cloned (*Bgl*II*/Bam*HI and *Avr*II) under control of the hp4d promoter in pYLHmAX (pYLHmA carrying the *URA3* selection marker) yielding pYLHmAXYlGIIa. To add the HDEL coding sequence to the ORFof GII α-subunt of *Y. lipolytica*, a PCR was performed on the obtained plasmid pYLHmAXYlGIIa with primers YlGlucIIαfw and YlGlucIIαHDELrv (Table 2), and the amplified fragment was cloned as described above for the version without HDEL tag.

#### Cloning the GII alpha-subunit of *Trypanosoma brucei* with and without HDEL tag

The ORF (2421 bp) of the GII α-subunit gene was amplified from genomic DNA of *T. brucei* (GenBank Accession No: AJ865333; a gift from Stijn Roge, Institute of Tropical Medicine, Antwerp) by PCR with primers TbGlucIIαfw and TbGlucIIαrv (Table 2) using *Pfu* DNA polymerase. The amplified fragment was cloned in pCR-Blunt-II-TOPO (Invitrogen, Carlsbad, CA, USA) and confirmed by sequencing. Next, it was subcloned *Bam*HI–*Avr*II in pYLHmAX, which contains the hp4d promoter and the *URA3* marker, yielding pYLHmAXTbGIIa. To add an HDEL tag to the *T. brucei* GII α-subunit, PCR was performed on the obtained plasmid with primers TbGlucIIαfw and TbGlucIIαHDELrv (Table 2), and the amplified fragment was cloned in the same way as without HDEL tag.

#### Cloning the GII beta-subunit of *Y. lipolytica*

The ORF (1288 bp) of the GII β-subunit gene was cloned from genomic DNA of *Y. lipolytica* MTLY60 (GenBank Accession No: XM_500467; Genolevures: YALI0B03652g) by PCR with primers YlGlucIIβfw and YlGlucIIβrv (Table 2) and *Pfu* DNA polymerase. Two other vectors (pYLHL and pYLTL) carrying the LEU2 selection marker were constructed for protein expression controlled by the hp4d or TEF promoter, respectively. Next, the ORF of *Y. lipolytica* GII β-subunit was cloned *Bam*HI–*Avr*II in these vectors, yielding pYLHLYlGIIb and pYLTLYlGIIb.

#### Cloning the GII alpha-subunit of *Aspergillus niger*

cDNA for a fusion of the ORF of the α-subunit of *A. niger* GII and an HDEL tag, flanked by *Sna*BI and *Avr*II, was synthesized by Geneart AG (Regensburg, Germany). The sequence was codon-optimized for expression in *Y. lipolytica*. First, two intermediate vectors were constructed, pYLTUXL2pre and pYLHUXL2pre, by introducing the pre sequence of LIP2 in pYLHmAX and pYLTmAX. The latter was derived from pYLHmAX by replacing the hp4d promoter by the TEF promoter. The introduction of the pre sequence of LIP2 was performed by annealing two primers (Table 2) and cloning them *Bam*HI-*Avr*II in pYLHmAX and pYLTmAX. The above-mentioned cDNA of the glucosidase α-subunit of *A. niger* flanked by *Sna*BI and *Avr*II was cloned in the corresponding restriction sites of pYLTUXL2pre and pYLHUXL2pre after *Sac*II digestion + T4 DNA polymerase blunting and *Avr*II digestion. The resultant plasmids (pYLTUXL2preAnGlucIIa and pYLHUXL2preAnGlucIIa, respectively) were confirmed by sequencing.

#### Cloning the GII beta-subunit of *A. niger*

The coding sequence for the β-subunit of *A. niger* GII flanked by *Eco*47III and *Avr*II restriction sites was synthesized by Geneart AG (Regensburg, Germany) as cDNA codon-optimized for expression in *Y. lipolytica*. Two intermediate vectors (pYLTLL2pre and pYLHLL2pre) were constructed by introducing the pre sequence of LIP2 in pYLTL and pYLHL, respectively, as described above. The resultant plasmids were named pYLTLL2pre and pYLHLL2pre. The above-mentioned synthesized cDNA was then cloned in the *Eco*47III and *Avr*II sites of these vectors by using *Sac*II digestion + T4 DNA polymerase blunting and *Avr*II digestion. The resultant plasmids (pYLTLL2preAnGlucIIb and pYLHLL2preAnGlucIIb, respectively) were confirmed by sequencing.

#### Cloning the *Trichoderma reesei* α-1,2-mannosidase with HDEL tag

We used an expression plasmid derived from pYLTUXL2preManHDEL [32] by digestion with I-*Sce*I followed by replacement of the *URA3* selection marker with the hygromycin selection marker (obtained from pKS-LPR-HYG, a gift from J.M. Nicaud, INRA) [52]. The resultant plasmid, pYLTHygL2preManHDEL, contains the *T. reesei* α-1,2-mannosidase coding sequence codon-optimized for *Y. lipolytica*, under control of a TEF promoter, preceded by the *Y. lipolytica* LIP2 pre signal sequence, and C-terminally tagged with an HDEL retrieval sequence.

#### Selection marker rescue

In all plasmids, the selection marker cassette is flanked by loxP and loxR sites to facilitate marker rescue by transient overexpression of the Cre recombinase. For overexpression of Cre recombinase, we used pRRQ2 (a gift from J.M. Nicaud, INRA) [52], which expresses the enzyme under control of the hp4d promoter and carries the *LEU2* resistance gene.

### Preparation of Mannoproteins, N-glycan Analysis and Exoglycosidase Digests

Yeast strains were inoculated and grown overnight in 10 mL of standard YPD medium in 50 mL Falcon tubes rotating at 250 rpm in a 28°C incubator. The cells were then pelleted at 4000 rpm in a cooled Eppendorf 5810R centrifuge. The supernatants were removed, and the cells were first washed with 2 mL of 0.9% NaCl solution followed by two washes with 2 mL of water and subsequently resuspended in 1.5 mL of 0.02 M sodium citrate pH 7 in an Eppendorf tube. After autoclaving for 90 min at 121°C, they were vortexed and the cellular debris was spun down. Then the supernatants were collected and the mannoproteins were precipitated overnight with four volumes of methanol at 4°C on a rotating wheel. After centrifugation, the pellets were allowed to dry and then dissolved in 50 µL of water.

The whole 50 µL of the cell wall protein solution was used to prepare N-glycans labeled with 8-aminopyrene-1.3.6-trisulphonic acid (APTS) according to a published method [55]. Then, fluorophore-assisted carbohydrate electrophoresis (FACE) was performed with an ABI 3130 DNA sequencer.

For the exoglycosidase digests, one tenth of the prepared APTS-labeled N-glycans was used. Exoglycosidase treatment of APTS-labeled glycans with Jack bean α-mannosidase (20 mU/digest, Sigma Biochemicals, Bornem, Belgium) or α-1,2-mannosidase (0.33 µg/digest, made in house) was performed overnight at 37°C in 50 mM ammonium acetate (pH 5.0). GII treatment of APTS-labeled glycans was performed with a purified rat liver mixture of alpha and beta (5 mU/mL, a gift from Dr. Terry Butters, Glycobiology Institute, Department of Biochemistry, Oxford, UK) [56]. Equal volumes of enzyme (in 80 mM triethylamine buffer, pH 7, containing 0.15 M NaCl and 10% glycerol) and sample were incubated together at 37°C overnight. The samples were then vacuum dried, resuspended in 10 µL of water, and analyzed on the ABI 3130 DNA sequencer.

### PNGaseF Treatment of Glycoproteins

Proteins in the *Yarrowia* culture medium were precipitated with two volumes of ice-cold acetone. After incubation on ice for 20 min and centrifugation at 14,000 rpm for 5 min, the supernatant was removed and the protein pellet was resuspended in 100 µL of 50 mM Tris-HCl, pH 8. SDS and β-mercaptoethanol were added to a final concentration of 0.5% and 1%, respectively. Samples were incubated for 5 min at 100°C, after which G7 buffer (10× buffer, New England Biolabs), NP-40 (final concentration of 1%), complete protease inhibitor (Roche) and in-house produced PNGaseF (15 IUBMB milliunits) were added. After overnight incubation at 37°C, proteins were precipitated by the deoxycholate/trichloroacetic acid (DOC/TCA) procedure, resuspended in 2× Laemmli buffer, and analyzed by SDS-PAGE.

### In vitro Digestion with *Trichoderma Harzianum* Mutanase

*T. harzianum* mutanase Novozyme 234, L1412 was obtained from Sigma-Aldrich Corporation, Spruce St., St. Louis, MO, USA. A stock solution of the enzyme (10 g/L) was prepared by dissolving 40 mg in 4 mL of 5 mM NH_4_Ac pH5 buffer. Five serial five-fold dilutions were made, and the final dilution (0.2 µL) was used to treat 0.5 µL of APTS-labeled N-glycans in a total volume of 10 µL buffered to a final concentration of 50 mM NH_4_Ac pH5. This reaction mixture was incubated overnight at 37°C and analyzed on an ABI 3130 DNA sequencer after desalting on a Sephadex G10 column [55].
